# Laser microdissection-based gene expression analysis in the aleurone layer and starchy endosperm of developing rice caryopses in the early storage phase

**DOI:** 10.1186/s12284-015-0057-2

**Published:** 2015-07-16

**Authors:** Tsutomu Ishimaru, Masashi Ida, Sakiko Hirose, Satoshi Shimamura, Takehiro Masumura, Naoko K. Nishizawa, Mikio Nakazono, Motohiko Kondo

**Affiliations:** NARO Institute of Crop Science, NARO, Kannondai, Tsukuba, Ibaraki 305-8518 Japan; Japan International Research Center for Agricultural Sciences, Ohwashi, Tsukuba, Ibaraki 305-8686 Japan; Graduate School of Life and Environmental Science Kyoto Prefectural University, Shimogamo, Sakyo-ku, Kyoto 606-8522 Japan; Graduate School of Agricultural and Life Sciences, University of Tokyo, Yayoi, Bunkyo, Tokyo 113-8657 Japan; Research Institute for Bioresources and Biotechnology, Ishikawa Prefectural University, 1-38 Suematsu, Nonoichi, Ishikawa 921-8836 Japan; Graduate School of Bioagricultural Sciences, Nagoya University, Furo, Chikusa, Nagoya 464-8601 Japan; International Rice Research Institute (IRRI), DAPO Box 7777, Metro Manila, Philippines; Life Science Research Institute, Kumiai Chemical Industry Co., Ltd., Shizuoka, 439-0031 Japan; National Institute of Agrobiological Sciences, Kannondai, Tsukuba, Ibaraki 305-8602 Japan; NARO Tohoku Agricultural Research Center (TARC), NARO, Kari-wano, Daisen, Akita 019-2112 Japan

**Keywords:** Aleurone cells, Early storage phase, Laser microdissection, *Oryza sativa* L, Rice, Starchy endosperm cells

## Abstract

**Background:**

Rice endosperm is composed of aleurone cells in the outermost layers and starchy endosperm cells in the inner part. The aleurone layer accumulates lipids, whereas starchy endosperm mainly accumulates starch. During the ripening stage, the starch accumulation rate is known to be asynchronous, depending on the position of the starchy endosperm. Different physiological and molecular mechanisms are hypothesized to underlie the qualitative and quantitative differences in storage products among developing rice endosperm tissues.

**Results:**

Target cells in aleurone layers and starchy endosperm were isolated by laser microdissection (LM), and RNAs were extracted from each endosperm tissue in the early storage phase. Genes important for carbohydrate metabolism in developing endosperm were analyzed using qRT-PCR, and some of the genes showed specific localization in either tissue of the endosperm. Aleurone layer-specific gene expression of a sucrose transporter, *OsSUT1*, suggested that the gene functions in sucrose uptake into aleurone cells. The expression levels of ADP-glucose pyrophosphorylase (*AGPL2* and *AGPS2b*) in each endosperm tissue spatially corresponded to the distribution of starch granules differentially observed among endosperm tissues. By contrast, expressions of genes for sucrose cleavage—hexokinase, UDP-glucose pyrophosphorylase, and phosphoglucomutase—were observed in all endosperm tissues tested. Aleurone cells predominantly expressed mRNAs for the TCA cycle and oxidative phosphorylation. This finding was supported by the presence of oxygen (8 % concentration) and large numbers of mitochondria in the aleurone layers. In contrast, oxygen was absent and only a few mitochondria were observed in the starchy endosperm. Genes for carbon fixation and the GS/GOGAT cycle were expressed highly in aleurone cells compared to starchy endosperm.

**Conclusions:**

The transcript level of *AGPL2* and *AGPS2b* encoding ADP-glucose pyrophosphorylase appears to regulate the asynchronous development of starch granules in developing caryopses. Aleurone cells appear to generate, at least partially, ATP via aerobic respiration as observed from specific expression of identified genes and large numbers of mitochondria. The LM-based expression analysis and physiological experiments provide insight into the molecular basis of the spatial and nutritional differences between rice aleurone cells and starchy endosperm cells.

**Electronic supplementary material:**

The online version of this article (doi:10.1186/s12284-015-0057-2) contains supplementary material, which is available to authorized users.

## Background

Endosperm accumulates large amounts of nutrients, including starch, proteins, lipids, and minerals, during the ripening stage. Rice (*Oryza sativa* L.) is the staple food of nearly half of the world’s population (Carriger and Vallee 2007). Rice endosperm is used not only as a source of carbohydrate (mainly starch) energy in the form of steamed rice, but also as a source of oils.

Rice endosperm consists of aleurone cells and starchy endosperm. Lipid is accumulated in aleurone cells, whereas starch is accumulated in starchy endosperm, starting at 5 days after flowering (DAF) (Hoshikawa 1967). Temporal changes in expression of genes for carbohydrate-metabolizing enzymes are closely associated with seed development and sugar status. At the pre-storage phase in rice, the ratio of hexose to sucrose is high, but it starts decreasing with the onset of the storage phase (Ishimaru et al. 2005). Sucrose is the dominant sugar transported into endosperm in the storage phase. Concomitantly, genes important in starch accumulation begin to be expressed at 5 DAF with the onset of starch accumulation. Sucrose is apoplastically unloaded into endosperm from maternal tissues by sucrose transporters. The rice sucrose transporter gene family comprises five genes, *OsSUT1-5* (Aoki et al. 2003). Among these five *OsSUT* genes, *OsSUT1* is expressed after 5 DAF (Hirose et al. 1997; Hirose et al. 2002) in aleurone cells in developing endosperm (Furbank et al. 2001; Ishimaru et al. 2007) and plays a critical role in starch accumulation in endosperm (Scofield et al. 2002). After uptake by a sucrose transporter, sucrose is metabolized by sucrose-cleavage enzymes including cell wall invertase and sucrose synthase. These enzymes are crucial for development, growth, and carbon partitioning to sink organs in plants (Sturm and Tang 1999). In rice, eight genes encoding cell wall invertase have been identified, with only *OsCIN2* expressed in developing endosperm (Cho et al. 2005). Functional analysis of a rice *grain incomplete filling 1* (*GIF1*) mutant has revealed that the cause is a single mutation in *OsCIN2* (Wang et al. 2008). For sucrose synthase, six genes have been identified and spatio-temporal expression in organs has been well characterized. *SUS3* and *SUS4* are expressed predominantly in developing grains, indicating potential roles in carbon allocation in filling grains (Hirose et al. 2008). Hexokinase (HXK), UDP-glucose pyrophosphorylase, and phosphoglucomutase (PGM) act at an intermediate metabolic step between sucrose cleavage and starch biosynthesis. Ten HXK genes have been cloned, of which five, *HXK2*, *HXK4*, *HXK5*, *HXK6,* and *HXK8*, are expressed at a higher level in endosperm than in pericarp, suggesting their role in endosperm (Cho et al. 2006). Two isoforms of UDP-glucose pyrophosphorylase, *OsUgp1* and *OsUgp2*, have been cloned in rice (Chen et al. 2007). Inactivation of *OsUgp1* causes grain chalkiness in addition to genic male sterility (Koh et al. 1999; Woo et al. 2008). All of the genes associated with starch biosynthesis, including ADP-glucose pyrophosphorylase (Ohdan et al. 2005), plastid translocator (Toyota et al. 2006), starch synthase (Hirose and Terao 2004), branching enzyme, starch debranching enzyme, phosphorylase, and disproportionating enzyme (Ohdan et al. 2005) have been cloned, and some of them are expressed concomitantly with the onset of endosperm starch accumulation at 5 DAF (Hirose and Terao 2004; Ohdan et al. 2005; Toyota et al. 2006). Genetic analyses using mutants and gene manipulation of starch biosynthesis-related genes have revealed the critical role(s) of some genes in grain phenotypes and starch properties in rice endosperm (Lee et al. 2007; Fujita et al. 2006; Fujita 2014; Umemoto et al. 2004; Fujita et al. 2007; Ryoo et al. 2007; Itoh et al. 2003; Satoh et al. 2003; Satoh et al. 2008; Nishi et al. 2001).

Histological studies have revealed the time course of development of storage product in rice developing endosperm. Aleurone cells begin their differentiation at 4–5 DAF in the outermost endosperm, whereas active starch accumulation starts in the center of the endosperm at 5 DAF (Hoshikawa 1968; Ishimaru et al. 2003). Starch accumulation proceeds asynchronously depending on the region. During the early storage phase, starch rapidly accumulates around the center of the endosperm, whereas in the peripheral starchy endosperm, starch accumulation proceeds at a slower rate until the late storage phase (Hoshikawa 1968). Nitrogen and mineral contents are higher in peripheral endosperm layers corresponding to aleurone cells (Itani et al. 2002). Thus, storage products are quantitatively and qualitatively different depending on their position in the rice endosperm. In barley and maize, histochemical studies have revealed that the oxygen gradient in the endosperm tissues is associated with energy status and the accumulation of storage products. In barley kernels, oxygen-rich regions in the lateral and peripheral endosperm begin starch accumulation first in endosperm tissues under high-ATP conditions, whereas the hypoxic region in the inner endosperm accumulates starch at the later stage (Rolletschek et al. 2004). In developing maize kernels, the oil-storing embryo is in a high-O_2_ state with high levels of metabolites of glycolytic intermediates and the mitochondrial TCA cycle and with some pools of amino acids (Rolletschek et al. 2005). Thus, in maize and barley, oxygen status differs among endosperm tissues and is closely linked with the accumulation of storage products. However, the quantification of mRNAs of carbohydrate-metabolizing enzymes, described above as important genes for starch accumulation in rice endosperm, was performed using RNAs extracted from whole developing caryopses. No attempt has yet been made to elucidate the asynchronous development of starch among rice endosperm tissues at molecular level. In addition, the oxygen gradient in the developing endosperm is not yet known in rice.

To advance our understanding at the molecular level of positional differences in storage products (lipid and starch) and the starch-accumulating rate in developing rice endosperm, expression analysis by dissection of targeted endosperm tissues is desirable. However, manual dissection of specific tissues is difficult, because aleurone cells and starchy endosperm are structurally connected and very soft in the early storage phase. Laser microdissection (LM) is a powerful tool for isolating targeted individual cells from heterogeneous tissue viewed under a microscope, using an intense laser beam (Emmert-Buck et al. 1996). With LM, we previously succeeded in developing a method for obtaining high-quality RNA from developing rice endosperm, facilitating precise expression analysis (Ishimaru et al. 2007).

In the present study, LM was applied to dissect endosperm tissues at the early storage phase, 7 DAF, when the differentiation of aleurone layers and starchy endosperm is already distinct (Ishimaru et al. 2003) and the degree of starch accumulation varies with endosperm region (Hoshikawa 1968). Using RNAs extracted from each endosperm tissue, qRT-PCR analysis was performed to quantify the expression levels of genes for carbohydrate-metabolizing enzymes. In addition, oxygen concentrations were measured in the developing rice endosperm to determine whether O_2_ gradients are coupled with different metabolic pathways among endosperm tissues.

## Results

### Microscopic observation of starch granules and lipids in the endosperm

**Fig. 1:**
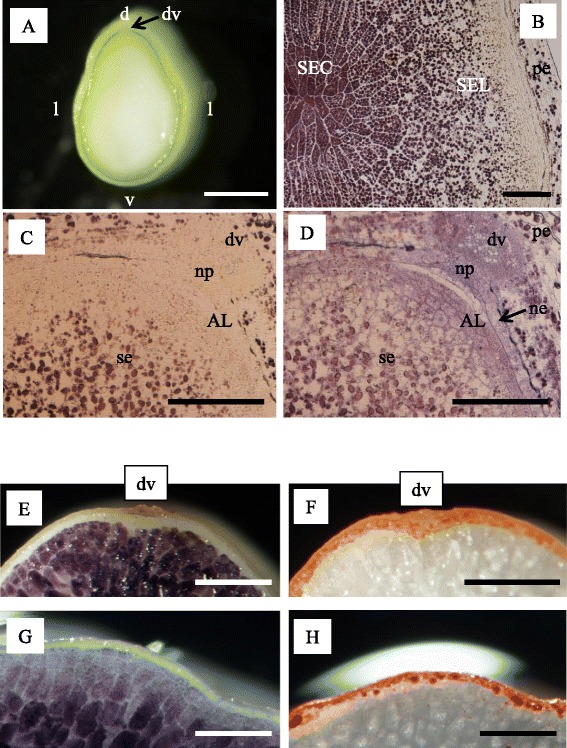
Observation of endosperm cells at 7 DAF (**a**–**d**) and in mature grain (**e**–**h**). **a** Median transversal section. **b** Microscopic observation of starchy endosperm at the center (SEC) and lateral side (SEL) stained with iodine. **c**, **d** Microscopic observation of endosperm at the dorsal side stained with iodine (**c**) and post-stained with toluindine blue-O (**d**). Matured grain stained with iodine (**e**, **g**) and Sudan IV (**f**, **h**). *AL* alurone cells, *d* dorsal side, *dv* dorsal vascular bundle, *l* lateral side, *ne* nucellar epidermis, *np* nucellar projection, *pe* pericarp, *v* ventral side. *Bar*: 1 mm (**a**), 200 μm (**b**–**d**), and 500 μm (**e**–**h**)

Under stereomicroscopic observation, the endosperm showed a uniform milky-white color (Fig. 1a), but morphology was qualitatively and quantitatively different among tissues observed with higher magnification. At 7 DAF, the starchy endosperm in the central region (SEC) accumulated much more starch than the starchy endosperm in the lateral regions (SEL) (Fig. 1b). In mature grain, iodine staining was lighter in SEL than in SEC (Fig. 1g). The surrounding outermost cell layer(s) were not stained with iodine solution (Fig. 1c, d, e, g). These cell layer(s) were identified as aleurone cells that were stained with Sudan IV with the accumulation of lipids at maturity (Fig. 1f, h). We defined the dorsal side of aleurone cells as AL (Fig. 1d).

### Transmission electron microscopic observation of endosperm cells

**Fig. 2:**
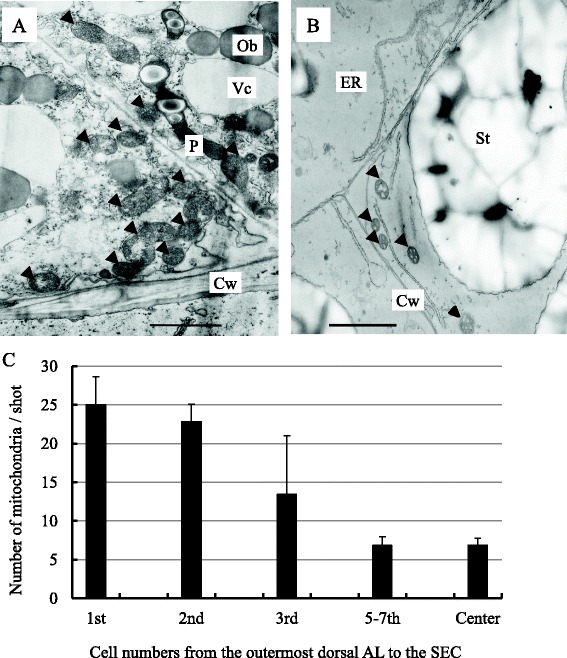
TEM observation of the outermost aleurone cell (**a**) and central starchy endosperm (**b**) and number of mitochondria along a dorso-ventral axis (**c**). *Mt* mitochondria, *St* starch, *ER* endoplasmic reticulum, *Vc* vacuole, *Cw* cell wall, *Ob* oil body, *P* proplastid. *Bar*, 2 μm

The development of organelles in endosperm cells at 7DAF was observed by transmission electron microscopy (TEM). Typical differences were observed in mitochondria in AL and in starch granules in endosperm cells (Fig. 2a and b). The mitochondria in the cells of developing endosperm were quantified along a dorso-ventral axis (the lateral regions of the endosperm are not included in this axis). In the first and second cell layers of the AL, approximately 25 mitochondria were observed per image (310.2 μm^2^; Fig. 2a, c), but mitochondrial density decreased by half in the third cell layer of the AL (Fig. 2c) and dropped to one fourth in the fifth cell layer of the starchy endosperm compared with the outermost AL cells (Fig. 2b, c). A similar number of mitochondria were observed below cell layers 5 to 7 (Fig. 2).

### Oxygen concentration of developing endosperm along a dorso-ventral axis

**Fig. 3:**
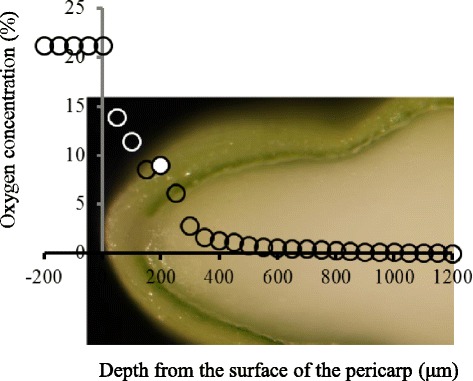
Oxygen concentration (%) profile along a dorso-ventral axis of a developing caryopsis at 7 DAF. The x-axis indicates the approximate position where the microelectrode was inserted. The *white enclosed circle* at 200-μm depth corresponds to the position of dorsal aleurone cells. An oxygen concentration profile was obtained from three kernels and values at each depth were averaged

In view of the differential distribution of mitochondria in the endosperm cell layers, oxygen concentration profiles along the dorso-ventral axis (SEL is not located on the x axis in Fig. 3) in developing rice caryopses at 7 DAF were investigated using a Clark-type O_2_ microelectrode according to the method of Shimamura et al. (2010). We found that the oxygen concentration decreased steeply as the microelectrode was being inserted deeper into the starchy endosperm. In particular, 8 % oxygen was observed at 200 μm from the surface of the caryopsis (the pericarp), a region corresponding to the dorsal side of AL (Fig. 3). When the electrode was inserted to a depth greater than 300 μm, where the microelectrode had already passed through the AL region and reached the inner starchy endosperm, oxygen concentration was lower than 2 % (Fig. 3). At 1200 μm, a region corresponding to the SEC, oxygen was absent.

### LM for endosperm cells at 7 DAF and identity confirmation of dissected tissues by qRT-PCR

**Fig. 4:**
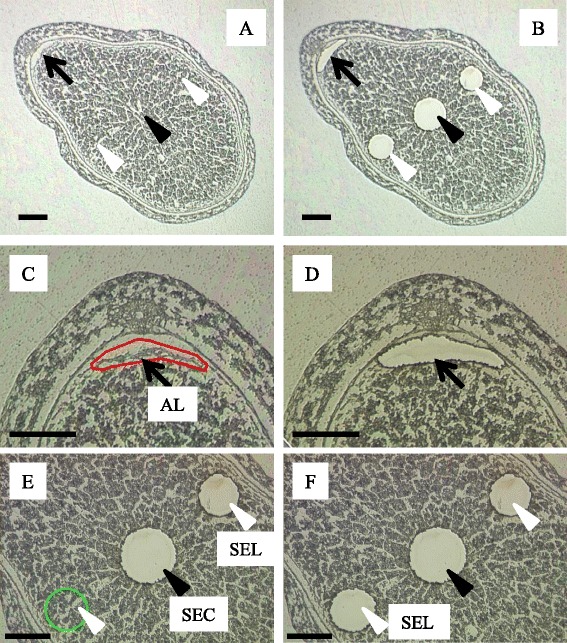
Microdissection of endosperm tissues. **a** Median transverse section. **b** Microdissection of aleurone cells from the dorsal side (*AL*; *black arrow*, magnified in **c** and **d**), starchy endosperm from the center region (*SEC*; *black arrowhead*, magnified in **e** and **f**) and lateral regions (*SEL*; *white arrowheads*, magnified in **e** and **f**). *Bar*: 200 μm

An overview of developing endosperm at 7 DAF is shown in Fig. 4a and b. AL (Fig. 4c, d), SEC (Fig. 4e), and SEL (Fig. 4e, f) were isolated by LM. We note that the aleurone cell layer at the outermost endosperm cells in the lateral regions was not isolated during dissection of the SEL (Fig. 4e, f). High-quality RNA, with RNA integrity number (RIN) > 7.0, was obtained from dissected tissues (Additional file 1: Table S1). The identities of dissected tissues were confirmed by qRT-PCR using primers for tissue-specific mRNAs for *oleosin*, 16 kDa isoform R16 (NCBI: AF022148), a structural protein found in oil bodies involved with lipid accumulation, and starch debranching enzyme (*SDBE*, Nakamura et al. 1996; NCBI: D50602), which specifically degrades amylopectin (Table 1). Expression values from quantitative RT-PCR were normalized to the transcript level of 18S rRNA (Kim et al. 2003; Additional file 2: Figure S1). The mRNAs for *oleosin*, 16 kDa isoform and *SDBE* were supposed to be markers specific for AL and starchy endosperm, respectively, because of the specific localization of starch granules and lipids in the endosperm (Fig. 1). Transcripts of *oleosin*, 16 kDa isoform R16, were detectable only in AL, whereas those of *SDBE* were at almost negligible levels in AL (Additional file 3: Figure S2A). The transcript level of *SDBE* was higher in the SEC than in the SEL (Additional file 3: Figure S2B) consistent with the gradients shown by iodine staining (Fig. 1b). These results confirmed the precise dissection of targeted endosperm tissues.

**Table 1:** Internal control and marker genes for aleurone cells and starchy endosperm

Category	NCBI accession number	Description	Reference
Internal control	X00755	18S rRNA	Kim et al. 2003
Marker for aleurone cells	AF022148	16 kDa oleosin isoform R16	NCBI^a^; Medina and Quatrano, unpublished.
Marker for starchy endosperm	D50602	Starch debranching enzyme	Pullulanase; Nakamura et al. 1996, OsPUL; Ohdan et al. 2005

^a^Direct submission to The National Center for Biotechnology Information (NCBI)

### qRT-PCR for genes associated with sucrose transport, sucrose cleavage, and starch biosynthesis

Transcription levels of genes associated with sucrose transport (Hirose et al. 1997), sucrose cleavage (cell wall invertase; Cho et al. 2005 and sucrose synthase; Hirose et al. 2008), hexokinase (Cho et al. 2006), UDP-glucose pyrophosphorylase (Chen et al. 2007), phosphoglucomutase (Akiyama et al. unpublished), and plastidis translocator (Toyota et al. 2006) and starch biosynthesis (Hirose and Terao 2004; Ohdan et al. 2005) during rice grain filling were evaluated. The selected genes are considered to play dominant roles in the developing endosperm among gene families with high expression or critical roles in grain phenotype and starch properties, according to previous genetic studies (see review by Fujita 2014, Table 2, and Background).

**Table 2:** Information on the genes related to sucrose transport, sucrose cleavage, intermediate metabolic steps and starch biosynthesis

Category	Accession No.	Description	Reference	Major changes in grain appearance and starch properties with the mutant and genetic manipulation of gene
Sucrose transport and cleavage	D87819	Sucrose transporter (SUT1)	OsSUT1; Hirose et al. 1997	Impaired grain filling (Scofield et al. 2002)
	AK072276	Cell wall invertase 2	OsCIN2; Cho et al. 2005	Chalky phenotype with abnormal amyloplast (gif1; Wang et al. 2008)
	AK100306	Sucrose synthase 3	SUS3; Hirose et al. 2008	-
	AK102158	Sucrose synthase 4	SUS4; Hirose et al. 2008	-
Metabolic step between sucrose cleavage and starch biosynthesis	DQ116384	Hexokinase 2	OsHXK2; Cho et al. 2006	-
	DQ116386	Hexokinase 4	OsHXK4; Cho et al. 2006	-
	DQ116387	Hexokinase 5	OsHXK5; Cho et al. 2006	-
	DQ116388	Hexokinase 6	OsHXK6; Cho et al. 2006	-
	DQ116390	Hexokinase 8	OsHXK8; Cho et al. 2006	-
	AB062606	UDP-glucose pyrophosphorylase	UGPase; Abe et al. 2002, OsUgp1; Chen et al. 2007, UGPase1; Woo et al. 2008	Chalky phenotype (Koh et al. 1999)
	AF455812	Phosphoglucomutase	NCBI^a^; Akiyama, unpublished.	-
Starch biosynthesis	U66041	ADP-glucose pyrophosphorylase large subunit	OsAGPL2; Ohdan et al. 2005; Lee et al. 2007	shrunken phenotype (Lee et al. 2007)
	AK103906	ADP-glucose pyrophosphorylase small subunit	OsAGPS2b; Ohdan et al. 2005; Lee et al. 2007	shrunken phenotype (Lee et al. 2007)
	AK107368	ADP-glucose transporter	OsBT1-1; Toyota et al. 2006	brittle phenotype (maize; Shannon et al. 1998, barley; Patron et al. 2004)
	D16202	Soluble starch synthase 1	SSS; Baba et al. 1993, SSI; Hirose and Terao 2004, OsSSI; Ohdan et al. 2005	Altered fine structure of amylopectin (Fujita et al. 2006)
	AF419099	Soluble starch synthase II-3	SSII-3; Hirose and Terao 2004, SSIIa; Ohdan et al. 2005	Altered fine structure of amylopectin (alk; Umemoto et al. 2004)
	AY100469	Soluble starch synthase III-2	SSIII-2; Hirose and Terao 2004, OsSSIII-2; Dian et al. 2005, OsSSIIIa; Ohdan et al. 2005	White-cored chalky phenotype and altered fine structure of amylopectin (Fujita et al. 2007, flo5; Ryoo et al. 2007)
	X62134	Granule-bound starch synthase I	Okagaki 1992, GBSSI; Hirose and Terao 2004, OsGBSSI; Ohdan et al. 2005	waxy phenotype with the absence of amylose (Itoh et al. 2003)
	D11082	Starch branching enzyme I	RBEI; Mizuno et al. 1992, OsBE1; Ohdan et al. 2005	Altered fine structure of amylopectin (sbeI; Satoh et al. 2003)
	D16201	Starch branching enzyme IIb	RBEIII; Mizuno et al. 1993, OsBEIIb; Ohdan et al. 2005	Chalky phenotype and altered fine structure of amylopectin (amylose-extender; Nishi et al. 2001)
	AB093426	Starch debranching enzyme: Isoamylase I	OsISA1; Ohdan et al. 2005	sugary phenotype (sugary-1; Kubo et al. 1999a)
	AK063766	Plastidial phosphorylase	OsPHOL; Ohdan et al. 2005	shrunken to pseudonormal phenotypes (pho1; Satoh et al. 2008), protein phosphorylation in amyloplast (wheat; Tetlow et al. 2004)

^a^Direct submission to The National Center for Biotechnology Information (NCBI)

**Fig. 5:**
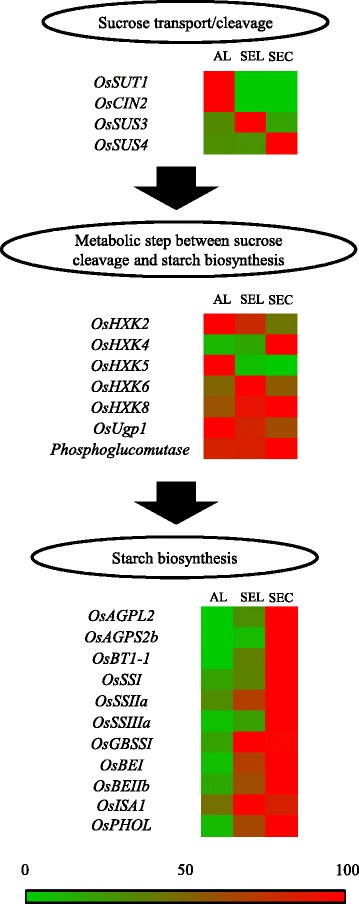
qRT-PCR analysis of sucrose transport, sucrose cleavage, intermediate metabolic steps and starch biosynthesis. Values are shown in a sequential color chart from 0 (*green*) to 100 (*red*). The tissue with the highest value among endosperm tissues was adjusted to 100. The values are means of three biological replicates. The accession numbers and primer pairs for each gene are given in Table 2

The expression of *OsSUT1* was specific to aleurone cells and undetectable in the SEL and SEC. The expression levels were contrasting among the genes for sucrose-cleavage enzymes. The highest expression levels for *OsCIN2*, *OsSUS3*, and *OsSUS4* were observed in AL, SEL, and SEC, respectively (Fig. 5).

A major role of hexokinase (*OsHXK*) genes in rice developing endosperm has not yet been clarified by genetic approaches, but the genes *OsHXK2*, *OsHXK4*, *OsHXK5*, *OsHXK6*, and *OsHXK8* were selected in view of their preferential expression in developing endosperm, based on the report of Cho et al. (2006). *OsHXK4* and *OsHXK5* were expressed predominantly in SEC and AL, respectively. The expression of *OsHXK2* and *OsHXK6* was observed in all tissues, but preferentially in AL and SEL, respectively. The expression of *OsHXK8* was higher in SEL and SEC compared to AL. Expression of *OsUgp1* and *phosphoglucomutase* was observed in all tissues.

The expression of genes for starch biosynthesis was variable. Expression was at low level (with values below 25) in AL for all genes except *OsSSIIa* and *OsISA1*. The expression of *OsAGPL2* and *OsAGPS2b*, *OsBT1-1*, *OsSSI*, *OsSSIIIa*, and *OsPHOL* was preferential in SEC. The expression of genes for *OsSSIIa*, *OsBEI*, and *OsBEIIb* was relatively high (with values over 60) in the SEL and highest in the SEC. The expression of genes for *OsGBSSI* and *OsISA1* was highest in SEL among endosperm tissues tested. We also investigated the expression of disproportionating enzyme (*OsDPE1*; Ohdan et al. 2005), which plays a critical role in building the amylopectin structure in Chlamydomonas reinhardtii (Colleoni et al. 1999). The expression of *OsDPE1* was undetectable in any tissues at 7DAF (data not shown), possibly because the transcript level abruptly decreases after 5DAF in the developing caryopsis (Ohdan et al. 2005).

### qRT-PCR of genes associated with CO_2_ fixation, the TCA cycle, oxidative phosphorylation, and the GS/GOGAT cycle

**Fig. 6:**
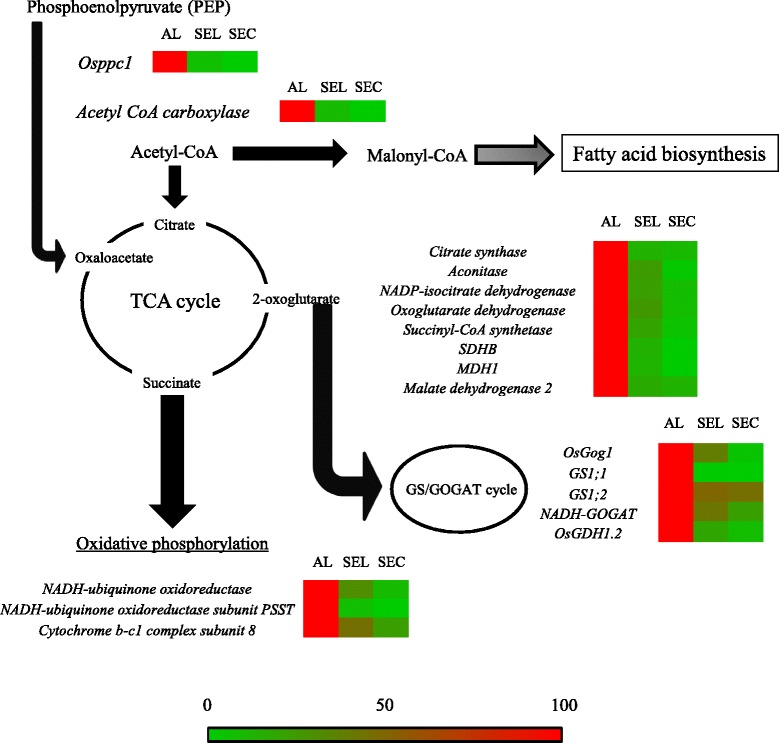
qRT-PCR analysis for carbon fixation, TCA cycle, oxidative phosphorylation, and GS/GOGAT cycle. Succinate dehydrogenase is also classified as a gene for oxidative phosphorylation. The sequential color chart refers to Fig. 4. The accession numbers and primer pairs for each gene are given in Table 3

The presence of oxygen (8 %) in the AL (Fig. 3), suggested the high expression of genes associated with aerobic respiration. In addition to the expression of genes for TCA cycle and oxidative phosphorylation, the expression of genes associated with CO_2_ fixation and the GS/GOGAT cycle, a metabolic step is close to the TCA cycle and oxidative phosphorylation, was evaluated (Table 3; Fig. 6). Some of the selected genes have not yet been characterized, but they appear in NCBI (http://www.ncbi.nlm.nih.gov/nuccore/) and RAP-DB (http://rapdb.dna.affrc.go.jp/) as our target genes based on their sequence similarity to genes in other plant species (Table 3).

**Table 3:** Information on the genes related to carbon fixation, TCA cycle, oxidative phosphorylation and GS/GOGAT cycle

Category	Accession No.	Description	Reference
Carbon fixation	AK059261	Acetyl CoA carboxylase	RAP-DB^a^; Similar to Acetyl-coenzyme A carboxylase. (Os05t0295300-01)
	AF271995	Phosphoenolpyruvate carboxylase 1	Osppc1; Yamamoto et al. 2014
TCA cycle	AF302906	Citrate synthase	NCBI^b^; Silva et al. Unpublished.
	AK067183	Aconitase	RAP-DB^a^; Similar to Aconitate hydratase, cytoplasmic (Citrate hydro-lyase) (Aconitase). (Os03t0136900-01). Similar to Aconitate hydratase 2, mitochondrial. (Os03t0136900-02)
	AF155333	NADP-isocitrate dehydrogenase	Koyama et al. 1999
	AK100482	Oxoglutarate dehydrogenase	RAP-DB^a^; Similar to 2-oxoglutarate dehydrogenase, E1 component. (Os07t0695800-01)
	AK103525	Succinyl-CoA synthetase	RAP-DB^a^; Similar to Succinyl-CoA synthetase, beta chain. (Os02t0621700-01)
	AB017428	Succinate dehydrogenase	SDHB; Kubo et al. 1999b
	AF444195	Malate dehydrogenase 1	MDH; Lin et al. 2003
	AK073698	Malate dehydrogenase 2	RAP-DB^a^; Similar to Malate dehydrogenase. (Os05t0574400-01)
Oxidative phosphorylation	AK058713	NADH-ubiquinone oxidoreductase	RAP-DB^a^; Similar to NADH-ubiquinone oxidoreductase 75 kDa subunit, mitochondrial precursor. (Os03t0713400-01); Similar to NADH-ubiquinone oxidoreductase 75 kDa subunit. (Os03t0713400-02); Similar to NADH-ubiquinone oxidoreductase 75 kDa subunit. (Os03t0713400-03)
	AK243655	NADH-ubiquinone oxidoreductase subunit PSST	RAP-DB^a^; NADH-ubiquinone oxidoreductase subunit PSST (Fragment). (Os05t0533700-01)
	AK119716	Cytochrome b-c1 complex subunit 8	RAP-DB^a^; Similar to Cytochrome b-c1 complex subunit 8. (Os06t0175900-01)
GS/GOGAT cycle	Y12594	Ferredoxin-dependent glutamate synthase (Fd-GOGAT)	OsGog1; Mattana et al. Unpublished^b^.
	X14245	Cytosolic glutamine synthetase 1;1	GS1; Sakamoto et al. 1989, GS1;1; Tabuchi et al. 2005
	X14244	Cytosolic glutamine synthetase 1;2	GSr; Sakamoto et al. 1989, GS1;2; Tabuchi et al. 2005
	AB008845	NADH dependent Glutamate Synthase	Goto et al. 1998
	AY332470	Glutamate dehydrogenase	OsGDH1.2; Qiu et al. 2009

^a^The Rice Annotation Project Database

^b^Direct submission to The National Center for Biotechnology Information (NCBI)

All the genes analyzed showed highest expression in AL, and their expression levels decreased in the order SEL > SEC (Fig. 6). Some of the genes were localized predominantly in the AL (*Osppc1*, *Acetyl-CoA carboxylase*, genes for the TCA cycle, *NADH-ubiquinone oxidoreductase subunit PSST*, *GS1;1*, *OsGDH1.2*).

## Discussion

### Transcripts associated with metabolic steps from sucrose transport to starch biosynthesis were differentially distributed in developing endosperm

Genetic analyses using mutants and gene manipulation have revealed the critical role(s) of genes for carbohydrate-metabolizing enzymes in kernel phenotypes and starch properties (Table 2). In this study, LM was applied to the AL, SEL and SEC (Fig. 4) to quantify the distribution of transcripts at metabolic steps from sucrose transport to starch biosynthesis, relative to their spatial distribution in starch granules among tissues in the early storage phase (Fig. 1).

The transcript of sucrose transporter 1 (*OsSUT1*; Hirose et al. 1997) was specific to AL, as we reported previously for AL and SEC (Ishimaru et al. 2007). This study showed negligible expression in SEL (Fig. 5). An anti-sense transformant of *OsSUT1* showed impaired grain filling (Scofield et al. 2002). The specific localization of *OsSUT1* in AL (Fig. 5) suggests the critical function of aleurone cells for uptake of sucrose into developing endosperm tissues for starch accumulation. Our LM-based expression analysis revealed the specific localization of *OsCIN2* in AL among endosperm tissues as that of *OsSUT1* (Fig. 5). A mutation of *OsCIN2* (*gif1*) causes slower grain filling and chalky phenotype of the kernel with aberrant amyloplast formation, indicating that hexoses catalyzed by *OsCIN2* function as an important carbon energy source for grain development in rice (Wang et al. 2008). The specific distribution of *OsCIN2* in AL (Fig. 5) was spatially inconsistent with starch accumulation in the starchy endosperm, such as in SEL and SEC. *OsCIN2* in AL is expected to contribute to the partitioning of hexoses to the inner endosperm tissues for starch accumulation in SEL and SEC. The organ expression analysis of *OsSUS3* and *OsSUS4* revealed their predominant localization in the developing caryopsis, especially in the endosperm, after the onset of starch accumulation at 5 DAF (Hirose et al. 2008). Our LM-based expression analysis found *OsSUS3* and *OsSUS4* to be localized mainly in the SEL and SEC, respectively (Fig. 5). The expression profile of the sucrose-cleavage genes *OsCIN2*, *OsSUS3*, and *OsSUS4* was spatially complemented in the developing endosperm. The cleavage of sucrose in the developing endosperm is necessary for generating a sucrose gradient to maintain sink strength (Sturm and Tang 1999). The results suggest that *OsCIN2*, *OsSUS3*, and *OsSUS4* contribute to sink strength by cleaving sucrose in different locations in developing endosperm tissues. With respect to the expression profile for hexokinase, *OsHXK4* and *OsHXK5* showed predominant expression in SEC and AL, respectively. For *OsHXK2*, a gradient in expression level was observed from AL to SEC in descending order. *OsHXK6* and *OsHXK8* showed highest expression level in SEL and SEC, respectively, and relatively high expression was observed remaining two tissues. Transcripts of *OsUgp1* and *phosphoglucomutase* were highest in AL and SEC, respectively, and relatively high expression was observed in the remaining two tissues. Thus, the expression of each gene appeared to be functional in a different space in hexokinase. *OsUgp1* and *phosphoglucomutase* are assumed to generate metabolites for subsequent starch biosynthesis in all three regions: AL, SEL, and SEC.

With respect to genes for starch biosynthesis, expression was low in AL, and the highest values, except for *OsGBSSI* and *OsISA1*, were observed in SEC (Fig. 5). Expression of *OsAGPL2*, *OsAGPS2b*, *OsBT1-1*, *OsSSI*, and *SSIIIa* was low in SEL, whereas expression of *OsSSIIa*, *OsGBSSI*, *OsBEI, OsBEIIb*, and *OsPHOL* was relatively high (with values over 60) in SEL. T-DNA insertion into either *OsAGPL2* or *OsAGPS2b* resulted in a shrunken phenotype of rice kernels with impaired grain filling (Lee et al. 2007). Mutant plants of *brittle-1* in maize (Shannon et al. 1998) and *lys5* in barley (Patron et al. 2004) show drastically decreased kernel dry weight, owing to the absence of a plastidial ADP-glucose transporter. The gradient of transcript abundance for *OsAGPL2*, *OsAGPS2b,* and *OsBT1-1* was consistent with the gradient of starch accumulation among AL, SEL, and SEC (Fig. 1), suggesting the in vivo regulation of starch accumulation by these genes at the transcriptional level. For starch synthase (both soluble and granule-bound type), starch branching enzymes, and starch debranching enzyme, greater attention has been paid to the effects of genes on starch properties such as amylopectin structure and amylose formation. Relatively high levels of expression of *OsSSIIa*, *OsGBSSI*, *OsBEI*, *OsBEIIb*, *OsISA1*, and *OsPHOL* in SEL suggest that these genes are responsible for the production of amylopectin and amylose in SEL at the transcriptional level. The subcellular location of *OsAGPL2* and *OsAGPS2b* is cytosolic (Sikka et al. 2001), whereas that of *OsBT1-1*, a gene for a starch synthase branching enzyme, and starch debranching enzyme is plastidial. In addition, the metabolic step of ADP-glucose pyrophosphorylase is located upstream from that of starch synthase, starch branching enzyme, and starch debranching enzyme. In SEL, the substrate of ADP-glucose may be deficient in plastids, owing to the very low level of cytosolic *OsAGPL2* and *OsAGPS2b*. Starch biosynthesis in the cereal endosperm is a complex process engaged with many genes (Fig. 5). Overall results of expression of genes associated with metabolic steps from sucrose transport to starch biosynthesis revealed the clear differences in the distribution of transcripts in the developing endosperm. The LM-based expression analysis conducted in this study provided the novel finding that the clear gradient of transcripts of *OsAGPL2* and *OsAGPS2b* may be responsible for the large difference in starch accumulation among AL, SEL, and SEC in the early storage phase.

### Aleurone cells are inferred to generate ATP by aerobic respiration during the early storage phase

Aleurone cells do not contain starch, but contain lipids (Fig. 1e and f). Our LM-based expression analysis showed the specific localization of *oleosin*, 16 kDa isoform R16, in AL (Additional file 3: Figure S2), and the low level of transcripts for starch biosynthesis in AL (Fig. 5). The expression analysis is consistent with the large amount of lipid and the absence of starch in AL (Fig. 1c). In developing maize, oxygen concentration is maintained at the high level in the oil-storing embryo, corresponding to the steady-state levels of glycolytic intermediates and those of the TCA cycle, as well as free amino acids (Rolletschek et al. 2005). In the present study, we investigated the spatial distribution of oxygen (Fig. 3) and transcripts of genes associated with carbon fixation, the TCA cycle, oxidative phosphorylation, and the GS/GOGAT cycle (Fig. 6). Oxygen was detectable in the outermost endosperm cells corresponding to the aleurone layers at 8 % (Fig. 3). All the genes examined in Table 3 were expressed dominantly in AL (Fig. 6), supporting the findings in maize embryo (Rolletschek et al. 2005) with respect to oxygen concentration and metabolites in oil-storage tissue. In the present study, we identified clear gradients in the number of mitochondria from the AL to the SEC in descending order (Fig. 2). Oparka et al. (1981) reported the presence of mitochondria in the aleurone and sub-aleurone layers with TEM observation. The present quantitative investigation showed that the profile of oxygen distribution agrees well with the gradient in numbers of mitochondria in rice endosperm in the early storage phase. Xu et al. (2008) reported that accumulation of proteins associated with the TCA cycle increased during 6–10 DAF (in the early storage phase), based on proteomic analysis of developing rice kernels. Thus, mitochondria localized in the oxygen-rich cells of AL are expected to contribute to ATP generation through aerobic respiration, thereby assisting the initial formation and accumulation of lipids via expression of genes listed in Table 3. In barley, starch accumulation is initiated in the lateral region, where the tissues retain a high level of oxygen supplied by photosynthesis in the surrounding pericarp (Rolleschek et al. 2004). In rice, starch accumulation is initiated in the center of the endosperm (Fig. 1b), where the tissue is most distant from the pericarp and in a condition of hypoxia (Fig. 3). Energy production for starch accumulation may be different between barley and rice, given the differences in oxygen availability in the starch-storing tissue in the early storage phase. SEC is assumed to produce ATP via anaerobic respiration to supply energy for starch development in the absence of oxygen (Fig. 3). Whether gradients in oxygen concentration are coupled with the differences in storage product between endosperm tissues (i.e. lipids in aleurone cells and starch in starchy endosperm) through the different process of energy production is still elusive. Further evidence are required to reveal the relationship between the energy production and biological process of storage product accumulation in the different positions of developing endosperm. Knockout mutants of cytosolic glutamine synthetase 1;1 showed a significant reduction in rice kernel weight (Tabuchi et al. 2005), indicating the involvement of this gene in carbon partitioning through nitrogen metabolism in the AL. The function of cytosolic glutamine synthetase 1;1 in the developing endosperm is still unclear, but there may be physiological linkages between nitrogen metabolism in aleurone cells and starch synthesis in starchy endosperm.

## Conclusions

We revealed the expression pattern of carbohydrate-metabolizing genes in the different positions of developing endosperm with an assistance of LM. The expression of *OsSUT1* was specific to the AL, and the expression of sucrose-cleavage enzymes such as *OsCIN1*, *OsSUS3*, and *OsSUS4* was preferential in AL, SEL and SEC, respectively. The gradients of transcript abundance for *OsAGPL2* and *OsAGPS2b* were assumed to be associated with the differential spatial distribution of starch granules among endosperm tissues in the early storage phase. These results in carbohydrate-metabolizing genes suggested the roles of each gene in carbon partitioning and starch synthesis in the different positions of developing endosperm. The presence of oxygen and large number of mitochondria in AL were consistent with the predominant expression of genes involved in TCA cycles and oxidative phosphorylation, inferring the energy production via aerobic respiration at least in part in AL. The LM-based expression analysis conducted in this study expanded molecular and physiological knowledge on the positional differences in starch accumulation and energy production in the developing rice endosperm in the early storage phase.

## Methods

### Plant materials

*Oryza sativa* cv. Koshihikari (a *Japonica* rice variety) was used. Seeds were sown in a nursery box filled with soil, and 4 weeks-old seedlings were transplanted into 0.02 m^2^ pots. As a basal dressing, 0.5, 2.3, and 2.2 g each of N, P_2_O_5_, and K_2_O was applied, and 0.4 g of nitrogen per pot was applied as a top dressing approximately two weeks before heading. At the booting stage, plants were transferred into a naturally illuminated temperature-controlled chamber. Day (13 h) and night (11 h) air temperatures were maintained at 26 and 20 °C, respectively until maturity. Spikelets were marked on the flowering day. Caryopses at 7 DAF located on the four primary rachis branches counted from the top of the panicles were used in all experiments.

### Laser microdissection (LM)

Four developing rice caryopses were collected from one panicle. Three biological replicates (panicles) were prepared from three rice plants. The preparation of specimens for LM followed Ishimaru et al. (2007). Briefly, the developing caryopses were immediately fixed with an ice-cold mixture of 3:1 ethanol: acetic acid, and embedded with 2 % carboxymethylcellulose. Transverse sections (8 μm thickness) were made at the median part of the developing caryopses with a cryomicrotome (Leica, CM1850). Aleurone cells at the dorsal side (AL) and starchy endosperm in the center (SEC) and the lateral regions (SEL) were microdissected with an AS LMD system (Leica Microsystems, Wetzlar, Germany).

### RNA extraction, quantification, and quality check

Total RNA was extracted with a Picopure RNA isolation kit (Molecular Devices, Sunnyvale, CA) using DNase I. Quantification of total RNA was determined by the fluorescence based method, using a RiboGreen RNA Quantification kit (Molecular Probes, Eugene, OR). The integrity of RNA from aleurone cells and starchy endosperm was assessed using a 2100 Bioanalyzer (Agilent technologies, Santa Clara, CA). The average RNA integrity number (RIN) with clear rRNA peaks from each tissue exceeded 7.0 for the three biological replications (Additional file 1: Table S1).

### cDNA synthesis and quantitative RT-PCR

Quantitative RT-PCR was performed following Ishimaru et al. (2009) with three biological replications. Total RNA (10 ng) was amplified and cDNA was synthesized with a WT-Ovation^TM^ RNA Amplification System (NuGEN technologies Inc., San Carlos, CA) according to the manufacturer’s instructions. For quantitative RT-PCR, SYBR Premix Ex Taq (TaKaRa Bio Inc, Shiga, Japan) was used with a real time RT-PCR system (7500 Real Time PCR System, Applied Biosystems, Foster, CA). Gene-specific primer pairs were designed with Primer 3 (version 0.4.0., http://frodo.wi.mit.edu/primer3/) or taken from previous reports (Additional file 4: Table S2). Dissociation curves confirmed the presence of a single amplicon in each PCR. cDNA of each gene was further amplified with Taq polymerase (ExTaq; TaKaRa Bio Inc), and PCR products were sequenced to confirm that the fragments were the targeted gene. For each gene, the highest value in a given tissue was adjusted to 100 after normalization with the transcript level of 18S rRNA (Kim et al. 2003; Additional file 2: Figure S1), and relative values less than 100 were determined for two other tissues.

### Oxygen concentration in developing caryopses

Oxygen concentration in developing caryopses was measured following Shimamura et al. (2010). Immediately after the detachment of the developing caryopsis from the glume, the caryopsis was placed in an aluminum block and fixed with dental silicone impression material (Provil novo light, Heraeus Kulzer GmbH, Germany). The base of the rachilla was covered with dental silicone impression material to prevent an inflow of external air after detachment. The impression material hardened within 2 min. A Clark-type O_2_ microelectrode (OX-25, Unisence A/S, Denmark) with a guard cathode and a tip diameter of 25 μm was inserted into a developing caryopsis at 50-μm intervals along a dorso-ventral axis. The microelectrode was connected to a pA meter (PA2000, Unisense A/S) and output was logged at 5-s intervals on a computer using an analog-to digital converter (ADC-16, Pico Technology, UK). The electrode was calibrated in air before measurement and in O_2_-free N_2_. The experiment was conducted at a temperature of 25 °C and 12 μmol m^−2^ s^−1^ photon flux density. Three developing caryopses sampled from different panicles were used. O_2_ concentrations obtained from three developing caryopses were averaged.

### Microscopic observation

#### Stereo microscope

Median transverse sections (1.0–1.5 mm thickness) of developing caryopses and matured grain were manually cut with a sharp razor. Sectioned mature kernels were immersed into a solution of 2 % KI and 0.4 % I_2_ or 2 % Sudan IV (*w/v*) in 70 % ethanol for staining starch or lipid, respectively. After staining, specimens were viewed under a stereomicroscope (SZX12, Olympus, Japan), and immediately photographed.

#### Light microscope

Developing caryopses were immersed in FAA (formalin: acetic acid: 70 % ethanol = 1: 1: 18), dehydrated in an ethanol series, and embedded in Technovit 7100 (Heraeus Kulzer GmbH, Hanau, Germany). Sections 3 μm thick were cut with a microtome (HM335E, Leica Microsystems, Germany). Sections were stained with a solution of 2 % KI and 0.4 % I_2_ to observe starch granules and the same specimens were then stained again with 0.1 % toluidine blue-O to observe median transverse endosperm.

#### Transmission electron microscope (TEM)

Developing caryopses at 7 DAF were hand-cut into 1.0 mm-thick sections at the median with a sharp razor and immediately fixed with ice-cold 4.0 % paraformaldehyde and 2.0 % glutaraldehyde for 3 h. Specimens were fixed again overnight at 4 °C. After washing with 100 mM phosphate buffer (pH 7.2), the specimens were post-fixed with 1 % osmium tetroxide overnight at 4 °C. They were then washed with distilled water, dehydrated in an ethanol series, and embedded in Spurr’s resin (Spurr Low Viscosity Embedding kit, Polyscience Inc., Warrington, PA). Sections 200 nm thick were cut with glass knives with an ultramicrotome (MT2-B, Sorvall, Newtown, CT) at the median part of the specimen. Sections were stained with TI blue (Nissin EM, Tokyo, Japan) for 2 h and with lead citrate for 7 min, and viewed under an H-7100 transmission electron microscope (Hitachi, Tokyo, Japan) at 75 kV. Mitochondria were counted from the images at ×3500 magnification on 76 × 50 mm films (310.2 μm^2^). Mean values were calculated with 13–29 independent images (cells) from two biological replications.

## Additional files

Additional file 1: Table S1. RNA integrity number (RIN) used for qRT-PCR analysis. Additional file 2: Figure S1. qRT-PCR for *18S rRNA.* The gene accession numbers and primer pairs are shown in Table 1. Values are the means of three biological replications. The value in each tissue was used for the normalization. Additional file 3: Figure S2. qRT-PCR for 16 kDa *oleosin* (A) and *starch debranching enzyme* (B; *SDBE*). The gene accession numbers and primer pairs are shown in Table 1. Values are the means of three biological replications. Additional file 4: Table S2. Gene-specific primers used in this study.
