# Supplementary material for: Laser microdissection-based gene expression analysis in the aleurone layer and starchy endosperm of developing rice caryopses in the early storage phase
Source: Rice (N Y). 2015 Jul 16;8:22. doi: 10.1186/s12284-015-0057-2 (PMC4503711; doi:10.1186/s12284-015-0057-2)
Supplement: Additional file 1: Table S1. — RNA integrity number (RIN) used for qRT-PCR analysis. [file 12284_2015_57_MOESM1_ESM.ppt]

## Slide 1
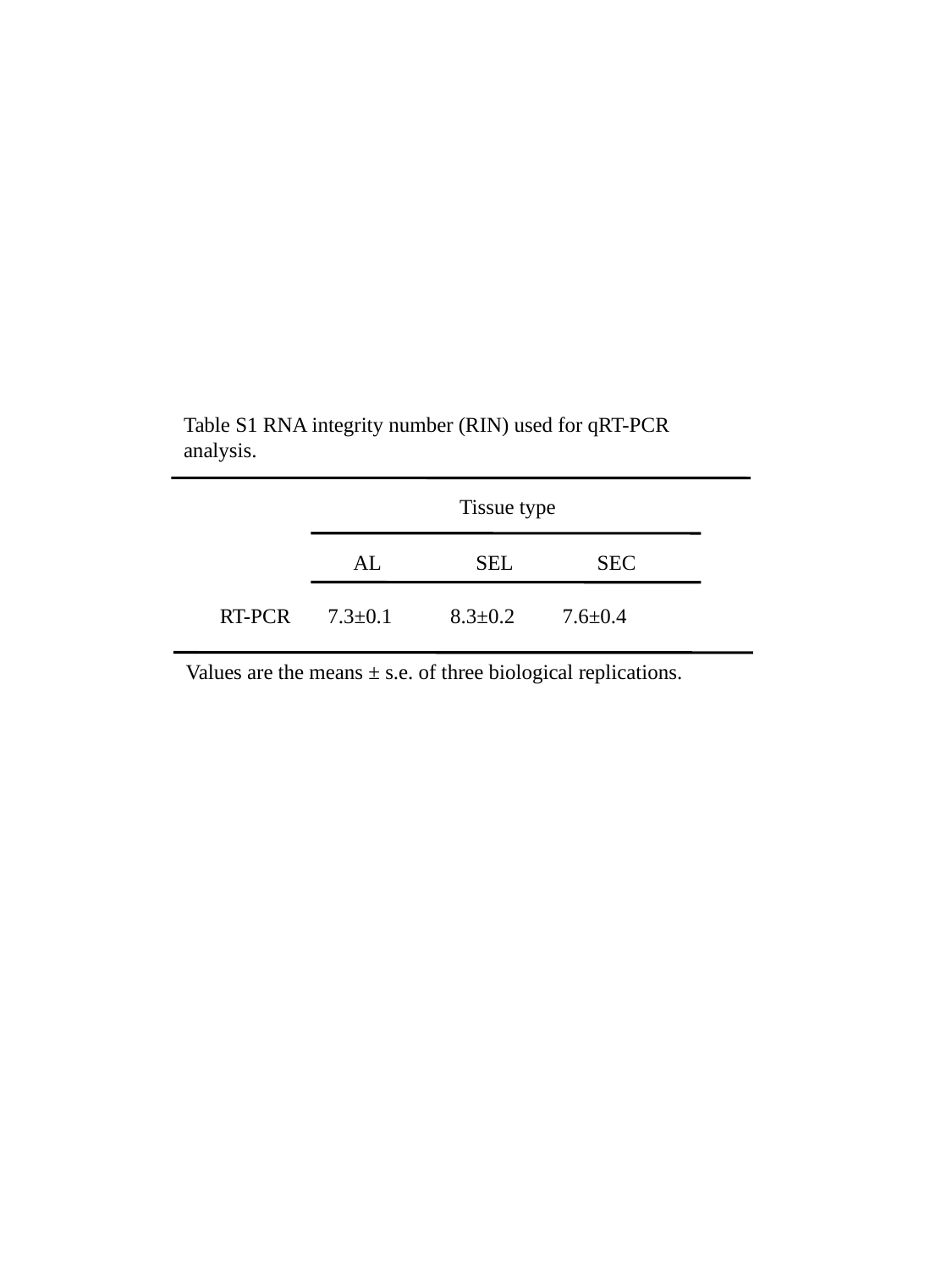

Table S1 RNA integrity number (RIN) used for qRT-PCR analysis.
Tissue type
AL SEL SEC
 RT-PCR 7.3±0.1 8.3±0.2 7.6±0.4
Values are the means ± s.e. of three biological replications.
