# Supplementary material for: Laser microdissection-based gene expression analysis in the aleurone layer and starchy endosperm of developing rice caryopses in the early storage phase
Source: Rice (N Y). 2015 Jul 16;8:22. doi: 10.1186/s12284-015-0057-2 (PMC4503711; doi:10.1186/s12284-015-0057-2)
Supplement: Additional file 2: Figure S1. — qRT-PCR for 18S rRNA. The gene accession numbers and primer pairs are shown in Table 1. Values are the means of three biological replications. The value in each tissue was used for the normalization. [file 12284_2015_57_MOESM2_ESM.ppt]

## Slide 1
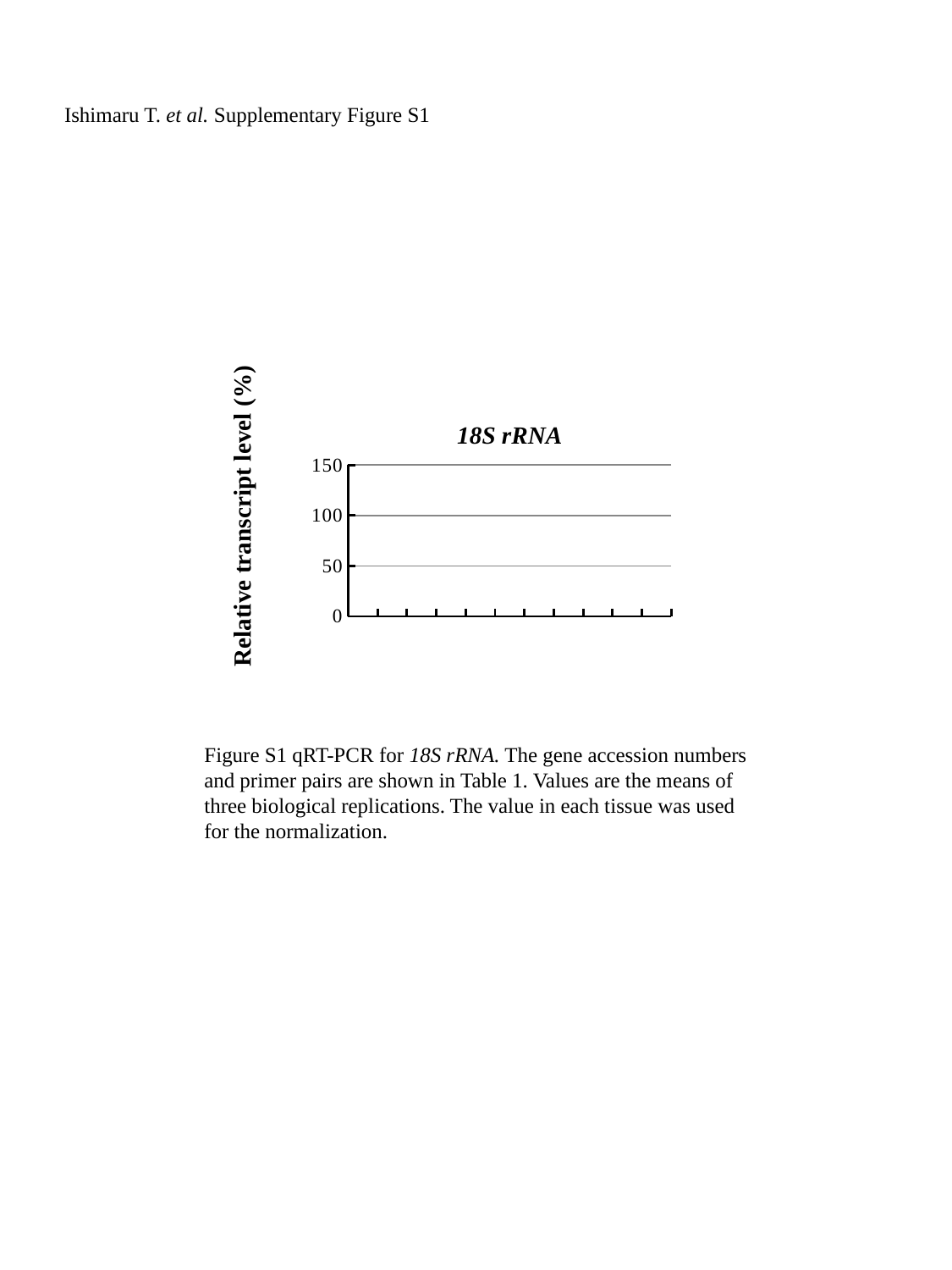

Ishimaru T. et al. Supplementary Figure S1
18S rRNA
### Chart
| Category | |
|---|---|Relative transcript level (%)
Figure S1 qRT-PCR for 18S rRNA. The gene accession numbers and primer pairs are shown in Table 1. Values are the means of three biological replications. The value in each tissue was used for the normalization.
