# Supplementary material for: Laser microdissection-based gene expression analysis in the aleurone layer and starchy endosperm of developing rice caryopses in the early storage phase
Source: Rice (N Y). 2015 Jul 16;8:22. doi: 10.1186/s12284-015-0057-2 (PMC4503711; doi:10.1186/s12284-015-0057-2)
Supplement: Additional file 3: Figure S2. — qRT-PCR for 16 kDa oleosin (A) and starch debranching enzyme (B; SDBE). The gene accession numbers and primer pairs are shown in Table 1. Values are the means of three biological replications. [file 12284_2015_57_MOESM3_ESM.ppt]

## Slide 1
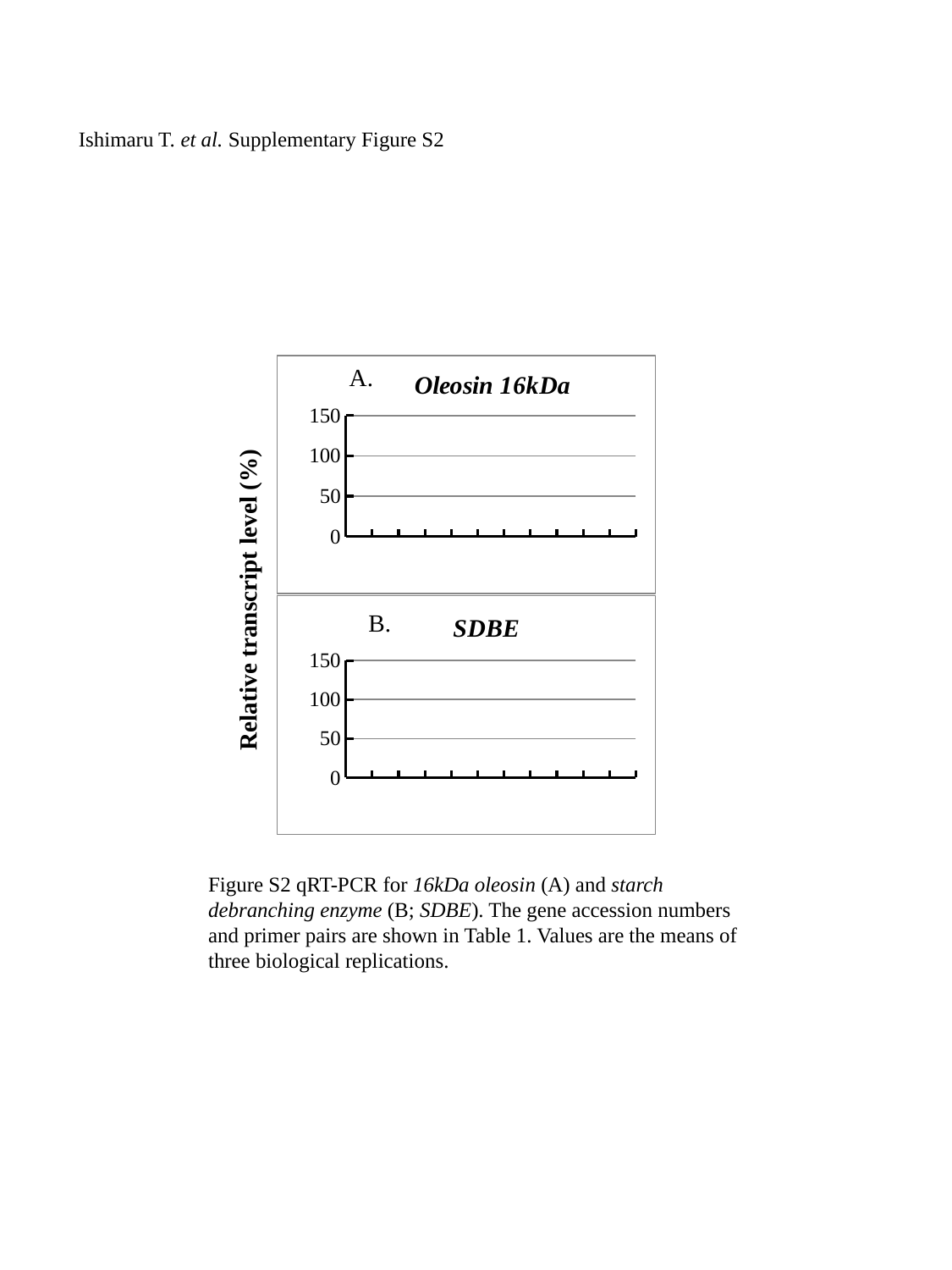

Ishimaru T. et al. Supplementary Figure S2
### Chart: Oleosin 16kDa
| Category | |
|---|---|A.
Relative transcript level (%)
### Chart: SDBE
| Category | |
|---|---|B.
Figure S2 qRT-PCR for 16kDa oleosin (A) and starch debranching enzyme (B; SDBE). The gene accession numbers and primer pairs are shown in Table 1. Values are the means of three biological replications.
