# Supplementary material for: Laser microdissection-based gene expression analysis in the aleurone layer and starchy endosperm of developing rice caryopses in the early storage phase
Source: Rice (N Y). 2015 Jul 16;8:22. doi: 10.1186/s12284-015-0057-2 (PMC4503711; doi:10.1186/s12284-015-0057-2)
Supplement: Additional file 4: Table S2. — Gene-specific primers used in this study. [file 12284_2015_57_MOESM4_ESM.ppt]

## Slide 1
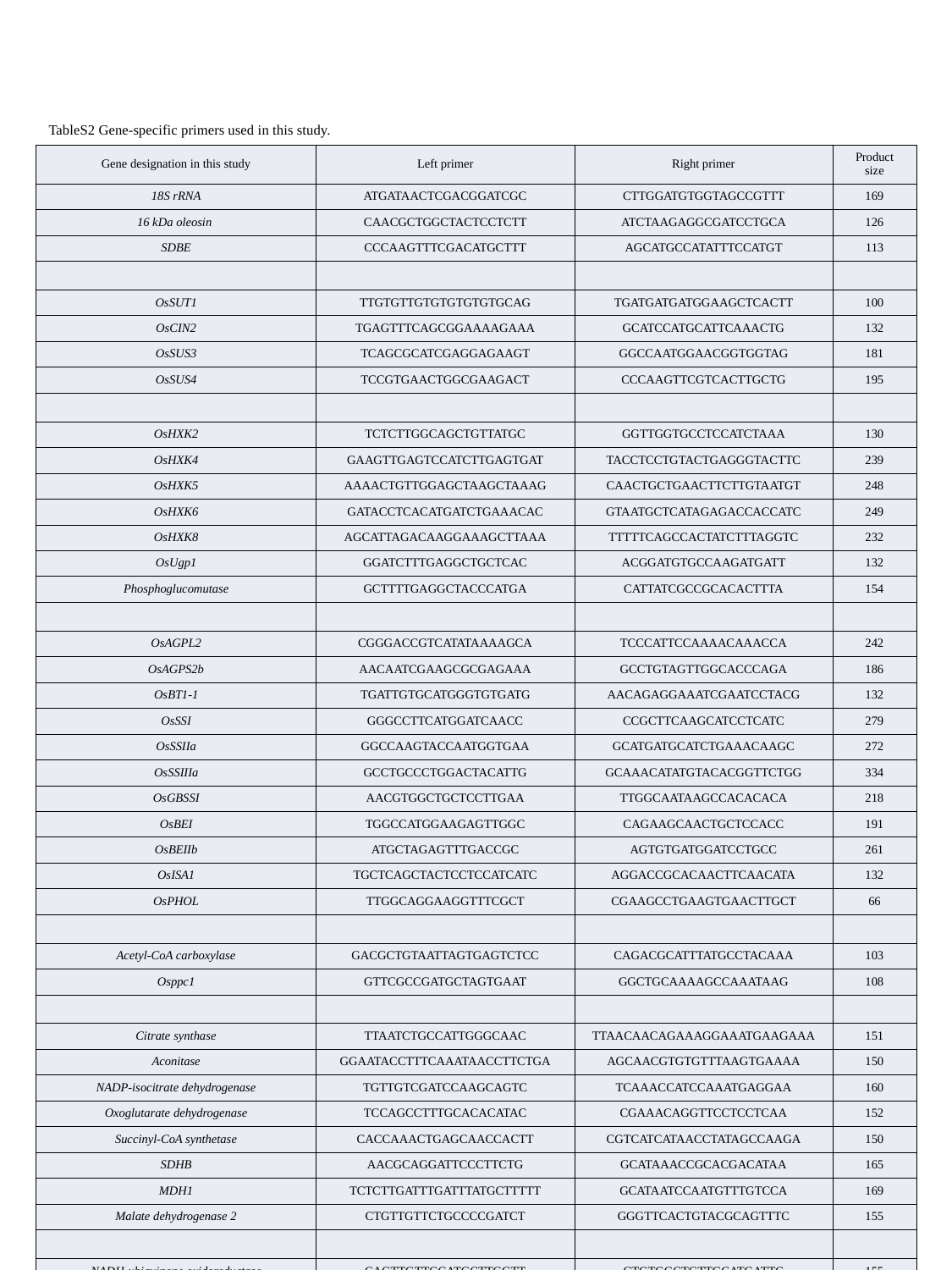

# TableS2 Gene-specific primers used in this study.
| Gene designation in this study | Left primer | Right primer | Product size |
| --- | --- | --- | --- |
| 18S rRNA | ATGATAACTCGACGGATCGC | CTTGGATGTGGTAGCCGTTT | 169 |
| 16 kDa oleosin | CAACGCTGGCTACTCCTCTT | ATCTAAGAGGCGATCCTGCA | 126 |
| SDBE | CCCAAGTTTCGACATGCTTT | AGCATGCCATATTTCCATGT | 113 |
| | | | |
| OsSUT1 | TTGTGTTGTGTGTGTGTGCAG | TGATGATGATGGAAGCTCACTT | 100 |
| OsCIN2 | TGAGTTTCAGCGGAAAAGAAA | GCATCCATGCATTCAAACTG | 132 |
| OsSUS3 | TCAGCGCATCGAGGAGAAGT | GGCCAATGGAACGGTGGTAG | 181 |
| OsSUS4 | TCCGTGAACTGGCGAAGACT | CCCAAGTTCGTCACTTGCTG | 195 |
| | | | |
| OsHXK2 | TCTCTTGGCAGCTGTTATGC | GGTTGGTGCCTCCATCTAAA | 130 |
| OsHXK4 | GAAGTTGAGTCCATCTTGAGTGAT | TACCTCCTGTACTGAGGGTACTTC | 239 |
| OsHXK5 | AAAACTGTTGGAGCTAAGCTAAAG | CAACTGCTGAACTTCTTGTAATGT | 248 |
| OsHXK6 | GATACCTCACATGATCTGAAACAC | GTAATGCTCATAGAGACCACCATC | 249 |
| OsHXK8 | AGCATTAGACAAGGAAAGCTTAAA | TTTTTCAGCCACTATCTTTAGGTC | 232 |
| OsUgp1 | GGATCTTTGAGGCTGCTCAC | ACGGATGTGCCAAGATGATT | 132 |
| Phosphoglucomutase | GCTTTTGAGGCTACCCATGA | CATTATCGCCGCACACTTTA | 154 |
| | | | |
| OsAGPL2 | CGGGACCGTCATATAAAAGCA | TCCCATTCCAAAACAAACCA | 242 |
| OsAGPS2b | AACAATCGAAGCGCGAGAAA | GCCTGTAGTTGGCACCCAGA | 186 |
| OsBT1-1 | TGATTGTGCATGGGTGTGATG | AACAGAGGAAATCGAATCCTACG | 132 |
| OsSSI | GGGCCTTCATGGATCAACC | CCGCTTCAAGCATCCTCATC | 279 |
| OsSSIIa | GGCCAAGTACCAATGGTGAA | GCATGATGCATCTGAAACAAGC | 272 |
| OsSSIIIa | GCCTGCCCTGGACTACATTG | GCAAACATATGTACACGGTTCTGG | 334 |
| OsGBSSI | AACGTGGCTGCTCCTTGAA | TTGGCAATAAGCCACACACA | 218 |
| OsBEI | TGGCCATGGAAGAGTTGGC | CAGAAGCAACTGCTCCACC | 191 |
| OsBEIIb | ATGCTAGAGTTTGACCGC | AGTGTGATGGATCCTGCC | 261 |
| OsISA1 | TGCTCAGCTACTCCTCCATCATC | AGGACCGCACAACTTCAACATA | 132 |
| OsPHOL | TTGGCAGGAAGGTTTCGCT | CGAAGCCTGAAGTGAACTTGCT | 66 |
| | | | |
| Acetyl-CoA carboxylase | GACGCTGTAATTAGTGAGTCTCC | CAGACGCATTTATGCCTACAAA | 103 |
| Osppc1 | GTTCGCCGATGCTAGTGAAT | GGCTGCAAAAGCCAAATAAG | 108 |
| | | | |
| Citrate synthase | TTAATCTGCCATTGGGCAAC | TTAACAACAGAAAGGAAATGAAGAAA | 151 |
| Aconitase | GGAATACCTTTCAAATAACCTTCTGA | AGCAACGTGTGTTTAAGTGAAAA | 150 |
| NADP-isocitrate dehydrogenase | TGTTGTCGATCCAAGCAGTC | TCAAACCATCCAAATGAGGAA | 160 |
| Oxoglutarate dehydrogenase | TCCAGCCTTTGCACACATAC | CGAAACAGGTTCCTCCTCAA | 152 |
| Succinyl-CoA synthetase | CACCAAACTGAGCAACCACTT | CGTCATCATAACCTATAGCCAAGA | 150 |
| SDHB | AACGCAGGATTCCCTTCTG | GCATAAACCGCACGACATAA | 165 |
| MDH1 | TCTCTTGATTTGATTTATGCTTTTT | GCATAATCCAATGTTTGTCCA | 169 |
| Malate dehydrogenase 2 | CTGTTGTTCTGCCCCGATCT | GGGTTCACTGTACGCAGTTTC | 155 |
| | | | |
| NADH-ubiquinone oxidoreductase | CAGTTGTTGCATGCTTGGTT | CTGTGGCTGTTGCATGATTC | 155 |
| NADH-ubiquinone oxidoreductase subunit PSST | GTGATGCTGTTGCGGTGTT | ATCATCACTTCTATTTACACAAGTTGG | 150 |
| Cytochrome b-c1 complex subunit 8 | TGTTTTCCATCGTTCCATGC | AAACAATTGCTTCAGTGACAGG | 102 |
| | | | |
| OsGog1 | CGTATTTTTGGTTCTGGACACA | CGATAACACCACGGTCCACT | 147 |
| GS1;1 | CGGTACTTGTCCCATCCTGT | CTATTGGAAGCCCAGCAAGA | 109 |
| GS1;2 | GTCCAACATGGACCCATACG | CAGAAATGCACGGGAGAGG | 102 |
| NADH -GOGAT | AGATAAGGCCGGGCTCAATA | TACAAAACGGCATTTCACCA | 134 |
| OsGDH1.2 | GGGTAGTGATGAATTGCCTGA | AGCCAAGAAACCAAATTCAGA | 120 |
